# Detection of Cd^2+^ based on Nano-Fe_3_O_4_/MoS_2_/Nafion/GCE sensor

**DOI:** 10.1007/s44211-023-00359-9

**Published:** 2023-06-05

**Authors:** Jiaqi Gao, Chengjun Qiu, Wei Qu, Yuan Zhuang, Ping Wang, Yirou Yan, Yuxuan Wu, Zexi Zeng, Gao Huang, Ruonan Deng, Guohui Yan, Jiaqi Yan, Ruoyu Zhang

**Affiliations:** 1 College of Mechanical, Naval Architecture and Ocean Engineering, Beibu Gulf University, Qinzhou, Guangxi China; 2 Guangxi Key Laboratory of Ocean Engineering Equipment and Technology, Qinzhou, China

**Keywords:** Nano-Fe_3_O_4_, MoS_2_, DPV, Cd^2+^

## Abstract

It is necessary to detect cadmium ions in seawater with high sensitivity because the pollution of cadmium ions seriously endangers the health and life of human beings. Nano-Fe_3_O_4_/MoS_2_/Nafion modified glassy carbon electrode was prepared by a drop coating method. The electrocatalytic properties of Nano-Fe_3_O_4_/MoS_2_/Nafion were measured by Cyclic Voltammetry (CV). Differential Pulse Voltammetry (DPV) was used to study the stripping Voltammetry response of the modified electrode to Cd^2+^. The optimal conditions were determined: In 0.1 mol/L HAc-NaAc solution, the solution pH was 4.2, the deposition potential was − 1.0 V, and the deposition time was 720 s, the membrane thickness was 8 μL. Under the optimum condition, the linear relation of Cd^2+^ concentration was found in the range of 5–300 μg/L, and the detection limit was 0.053 μg/L. The recovery of Cd^2+^ in seawater ranged from 99.2 to 102.9%. A composite material with simple operation, rapid response and high sensitivity was constructed for the determination of Cd^2+^ in seawater.

**Graphical abstract:**

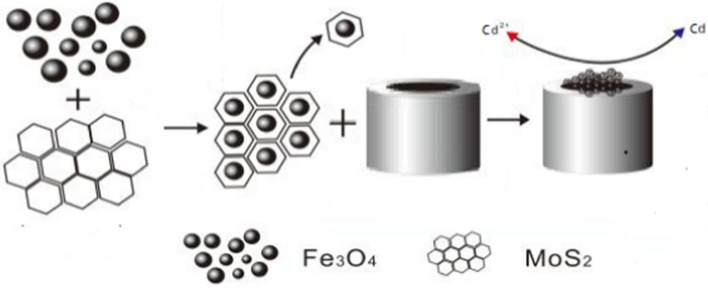

## Introduction

As a Category IA carcinogen [1], Cd^2+^ has carcinogenic [2], teratogenic and mutagenic effects [3], posing a serious threat to human life and health [4] and serious harm to the marine system [5]. The itai-itai disease caused by Japan is due to the discharge of wastewater from mining and smelting in Toyama Prefecture, Japan, which led to the accumulation of heavy metal Cd^2+^ in the river [6]. Finally, the Cd^2+^ directly or indirectly enters the human body [7], causing irreversible harm to the human body. Because of its harmfulness, the World Health Organization (WTO) and the United States Environmental Protection Agency (EPA) stipulate that the upper limit of Cd^2+^ in seawater shall be 5 μg/L [8]. These data suggest that it is necessary to address the pollution of cadmium ions (Cd^2+^) in marine ecosystems.

The current methods for detecting Cd^2+^ mainly include atomic absorption spectrometry (AAS) [9], atomic fluorescence spectrometry (AFS) [10], high-performance liquid chromatography (HPLC) [11] and ultraviolet spectrophotometry [12]. Although these detection methods are accurate and efficient [13], the instruments used are large, difficult to carry and cumbersome to operate. Therefore, electrochemical stripping voltammetry is used to detect Cd^2+^ in this paper. Compared with the previous methods, this method has simpler operation and lower cost. Moreover, it is convenient for real-time detection and is not limited to laboratory detection. In addition, electrochemical stripping voltammetry also has the advantages of high sensitivity and accurate detection [14]. The detection principle of the electrochemical stripping method is that Cd^2+^ is concentrated on the working electrode through electrodeposition, and then the analyte is oxidized and stripped by reverse voltage [15]. At a constant potential, the pre-enrichment reaction is:

1 $$ {\text{Cd}}^{{{2} + }} + 2{\text{e}}^{ - } \to {\text{Cd}}{.} $$

The electrode potential is scanned in the opposite direction, and the Cd^2+^ enriched on the electrode undergoes an oxidation reaction as follows:

2 $$ {\text{Cd + 2e}}^{ - } \to {\text{Cd}}^{{{2} + }} . $$

Nano-iron oxide (Nano-Fe_3_O_4_) has the advantages of strong electrical conductivity, strong adsorption capacity [16] and low price, but the bare Nano-Fe_3_O_4_ is very sensitive to redox reactions due to its high chemical reactivity and prone to agglomeration [17]. Molybdenum disulfide (MoS_2_), as a new type of two-dimensional nanomaterial, has three crystal structures, i.e., 1T, 2H and 3R. Among them, 2H has the most stable electrochemical performance, and at high temperature, both 1T and 3R crystal structures can be converted into 2H structures. Based on the stable electrochemical performance of 2H-MoS_2_ and its synergistic effect [18], this paper chooses MoS_2_ as the supporting matrix for Nano-Fe_3_O_4_. It can solve the problem of lower activity of MoS_2_ compared to noble metals [19], providing it with higher electrocatalytic activity. Nafion is a metal cation exchanger with antifouling properties. Its membrane is negatively charged, which can act as a natural barrier to prevent the interaction of negatively charged particles [20]. Based on the above factors, this paper uses Nano-Fe_3_O_4_, MoS_2_ and Nafion for composition to make a sensor that can detect Cd^2+^ ions rapidly with high sensitivity and solve the existing problems.

The process of making improvements to the electrode, The uniform suspension can be obtained by placing the Fe_3_O_4_ and MoS_2_ into Nafion (5 wt%) solution and then placing it into the ultrasonic cleaning machine for ultrasonic treatment. Then the suspension droplets are applied to the surface of the glassy carbon electrode (GCE) to obtain Fe_3_O_4_/MoS_2_/Nafion/GCE in.

In electrochemical detection, because electrode materials can effectively improve the detection performance of sensors, improving electrode materials is a hot spot in the research of electrochemical sensors. In the study of detecting Cd^2+^, there have been many electrochemical methods for detection, such as the use of MWCNTs/PAMT composites to detect Cd^2+^ in seawater and the use of GO/BiNPs/AuNPs composite materials for detection, although there are good results, but even so, the use of electrochemical methods to detect Cd^2+^ still has some problems, for example, due to the electrochemical activity of the material, although the sensitivity of Cd^2+^ is improved, but for the analysis speed of electrochemical system detection, The detection efficiency is greatly reduced, and improving the analysis speed, detection efficiency, will reduce the sensitivity of Cd, which is the current detection of Cd^2+^ need to solve the problem, and the Nano-Fe_3_O_4_/MoS_2_/Nafion detection Cd^2+^ used in this paper, because of the good electrochemical activity of Nano-Fe_3_O_4_, and the good stability of MoS_2_, the composite material obtained after the combination of the two, in the detection of Cd^2+^, can greatly improve the efficiency of detection and analysis speed, and can obtain better sensitivity, therefore, The use of Nano-Fe_3_O_4_/MoS_2_/Nafion energy will become a rapid and accurate detection and analysis of Cd^2+^ content in seawater, which has important academic value and social significance.

## Materials and methods

### Main instruments and reagents

The electrochemical experiment was carried out based on the CHI660 electrochemical analyzer of Shanghai Chenhua Instrument Co., Ltd. A three-electrode system was used, in which a 3 mm GCE was used as the working electrode and a platinum wire was used as the counter electrode. The saturated calomel electrode is the auxiliary electrode. Sigma HD scanning electron microscope (SEM) was used to observe the morphology of the modified electrodes. The polytetrafluoroethylene liner reactor, electronic analytical balance, suction filter device, ultrasonic cleaning machine, electric heating blast drying oven, and high-speed centrifuge are all from Shanghai Chenhua Instrument Co., Ltd.

The drugs used in the experiment were Na_2_MoO_4_·2H_2_O, CH_4_N_2_S, C_2_H_5_OH, FeCl_3_·6H_2_O, FeCl_2_·4H_2_O, NH_3_·H_2_O, CH_3_COOH, Nafion (5 wt%) solution, C_2_H_3_NaO_2_, Cd^2+^ standard solution and other reagents, all purchased from Sinopharm Chemical Reagent Co., Ltd. The purity used was either analytical reagent (AR) or guaranteed reagent (GR). The experimental water was ultrapure water with a resistivity of 18.0 MΩ cm. The actual water samples were taken from the seawater of the Maowei Sea in Qinzhou.

### Preparation of modified solution

An electronic analytical balance was used to accurately weigh 27.0296 g FeCl_3_·6H_2_O and 9.9415 g FeCl_2_·4H_2_O and pour them into a 300 mL round-bottom drying beaker (the molar ratio of Fe^2+^ to Fe^3+^ is 2:3), which was then added with ultrapure water to 250 mL and put on a magnetic stirrer. Then the solution was stirred until transparent. To deoxygenate the solution, the solution was injected with N_2_ for 30 min and stirred with a glass rod at a heating temperature of 50 °C. After stirring, NH_3_·H_2_O with a mass fraction of 15–18% was slowly added to adjust the pH of the mixed system to 8.0. Then the solution was placed at 75 ℃ for 1 h, after which the precipitate was separated with an external magnetic field, washed with ultrapure water and C_2_H_5_OH, and finally dried at 70 ℃ for 8 h to remove moisture and ground into powder -Fe_3_O_4_ powder.

An electronic analytical balance was used to accurately weigh 0.2420 g Na_2_MoO_4_·2H_2_O and 0.3806 g CH_4_N_2_S and pour them into a 100 mL round-bottom drying beaker, which was then added with ultrapure water to 60 mL. Then, 0.5 g prepared Nano-Fe_3_O_4_ was added, and the solution was poured into a polytetrafluoroethylene reaction kettle, which was heated for 18 h at 190 °C. Then, it was separated by a high-speed centrifuge, after which the collected black precipitate was washed several times with ultrapure water and C_2_H_5_OH and was dried in a drying oven at 80 ℃ for 10 h to obtain Nano-Fe_3_O_4_/MoS_2_ nanocomposite materials.

Finally, take 40 mg of the above materials, add 0.2 mL of absolute ethanol, 0.8 mL of ultrapure water and 80 μL of Nafion (5 wt%), ultrasound for 30 min can obtain Nano-Fe_3_O_4_/MoS_2_/Nafion modification solution. For comparative experiments, MoS_2_/Nafion solution without Nano-Fe_3_O_4_ was prepared by the same method.

### Preparation of modified electrodes

The GCE (diameter Φ = 3 mm) was polished with 1.0 μm, 0.3 μm and 50 nm alumina powders on a polishing cloth to obtain a bright surface, and then was rinsed with ultrapure water. After preliminary cleaning, it was put into an ultrasonic cleaner for several cleaning to remove the residual alumina powder on the surface. Finally, the GCE was put into a 0.5 mol/L H_2_O_4_ solution and scanned by cyclic voltammetry (CV) for several times at the potential of − 0.6 V to + 0.6 V until the curves were perfectly coincident. At this point, the electrode activation was completed. Next, 4 mg Nano-Fe_3_O_4_/MoS_2_ nano-composite was weighed and poured into a 10 mL round-bottom drying beaker, which was added with 80 μL Nafion (5 wt%) by a pipette and then added with 0.8 mL ultra-pure water and 0.2 mL C_2_H_5_OH. After the beaker was placed in an ultrasonic machine for 1 h, a uniform modified suspension solution of 4.0 mg/mL was obtained. Finally, an 8 μL modified solution was dropped onto the surface of the activated GCE using a pipette, and dried at room temperature to obtain the Cd^2+^ sensor.

### Experimental measurement

Cd^2+^ was detected by potentiostatic deposition and differential pulse voltammetry (DPV). First, CH_3_COOH and C_2_H_3_NaO_2_ were used to prepare 0.1 mol/L sodium acetate buffer solution (HAc-NaAc) with a pH of 4.2, which was used to dilute the Cd^2+^ standard solution to different concentrations. Next, a three-electrode system (the glassy carbon electrode was the working electrode, the Hg/HgCl electrode was the reference electrode, and the platinum wire electrode was the counter electrode) was used at room temperature. The Nano-Fe_3_O_4_/MoS_2_/Nafion/GCE, the platinum wire electrode and the Hg/HgCl electrode were inserted into the solution, and the Cd^2+^ was concentrated by potentiostatic deposition for 720 s at the potential of − 1.0 V under stirring condition. After the deposition was completed, the stirring was stopped. Finally, DPV detection was performed, in which the scanning potential interval was set to − 1.0 V to − 0.7 V, the potential increment was 50 mV, the frequency was 25 Hz, the amplitude was 5 mV and the rest of time was 2 s. Then the experiment was carried out to record the differential pulse voltammograms of different Cd^2+^ concentrations, draw the linear standard curve and calculate the detection limit. To ensure the accuracy of the experiment and avoid the interference caused by the previous deposition, after the completion of each experiment, it is necessary to increase the deposition potential by 0.5 V through the potentiostatic method, set the deposition time to 40 s, and perform constant potential deposition to remove the Cd^2+^ left over from the previous experiment on the GCE.

## Results

### SEM image analysis

**Fig. 1:**
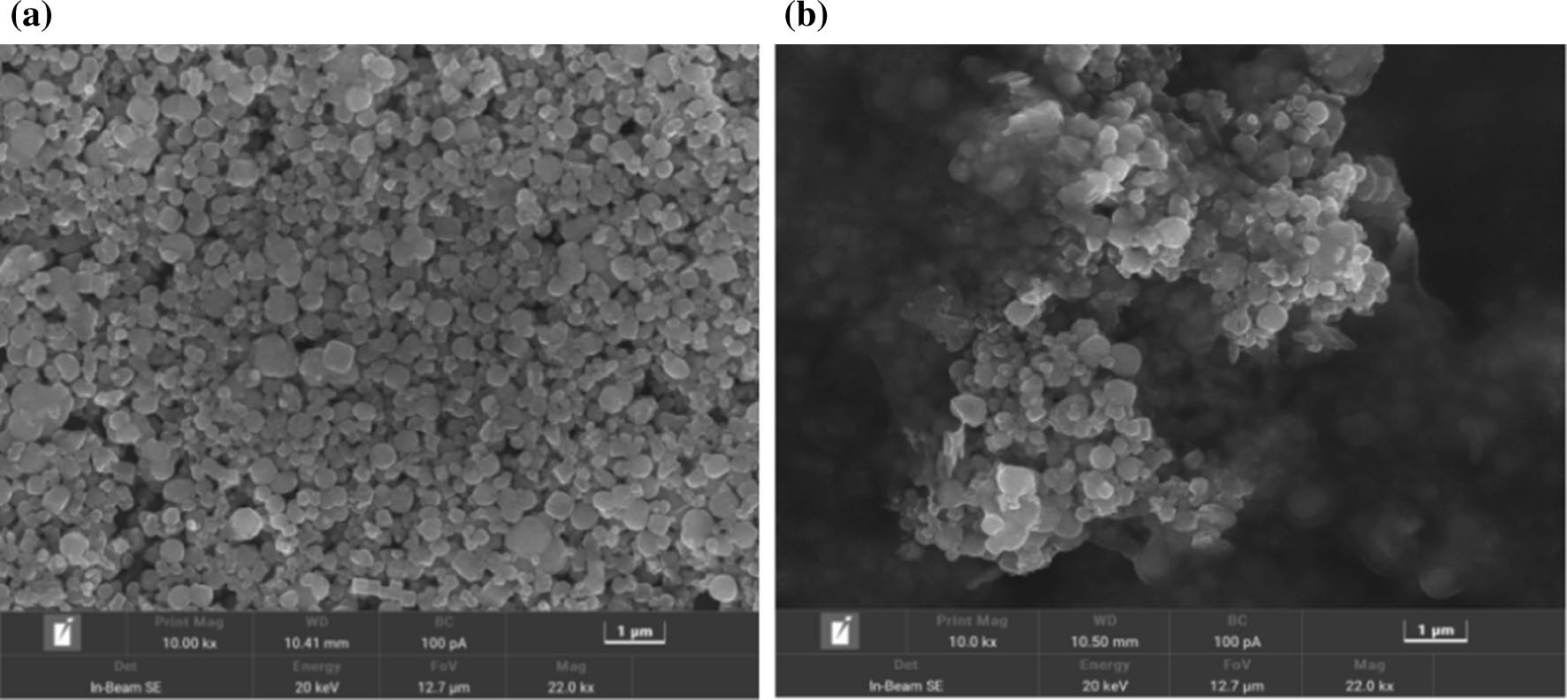
**a** SEM image of Nano-Fe_3_O_4_. **b** SEM image of Nano-Fe_3_O_4_/MoS_2_/Nafion

**Fig. 2:**
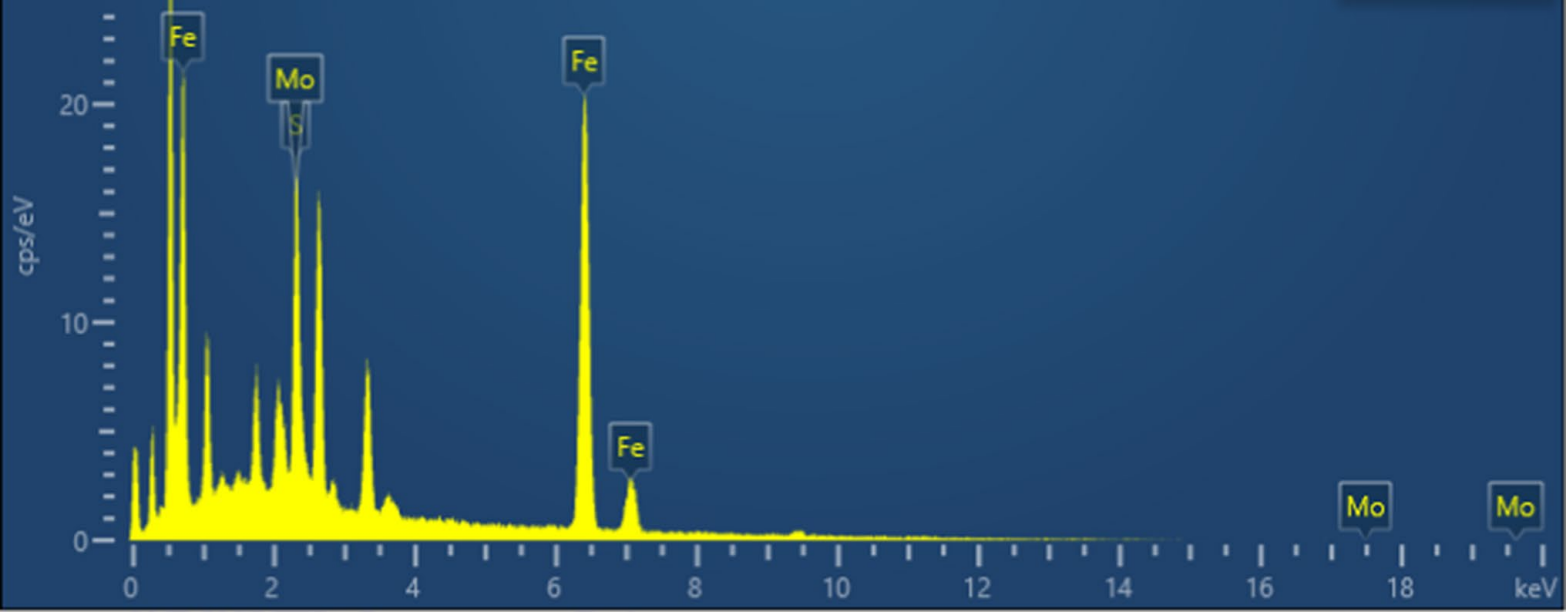
EDS mapping of Nano-Fe_3_O_4_/MoS_2_/Nafion

The morphological characteristics of Nano-Fe_3_O_4_ and Nano-Fe_3_O_4_/MoS_2_/Nafion composites were observed by scanning electron microscope. Figure 1(a) shows the microstructure of Nano-Fe_3_O_4_. under the magnification of Mag = 22 kx, Nano-Fe_3_O_4_ is spherical or nearly spherical, with uniform size. Figure 2(b) shows the microstructure diagram of Nano-Fe_3_O_4_/MoS_2_/Nafion, it is evident that MoS_2_ is entangled with Fe_3_O_4_ nanosheets to form an interconnected conductive network, which ensures optimal accessibility of electrolyte ions.

To further analyze the composite situation of the composite, EDS mapping was carried out to analyze the composite material, and the results are shown in Fig. 2, in the Nano-Fe_3_O_4_/MoS_2_/Nafion modification solution, The content of Fe is much higher than that of Mo and S, the percentage concentration of S element solution is 4.69 wt%, the percentage concentration of Fe solution is 78.41 wt%, and the percentage concentration of Mo is 16.9 wt%, in addition, Their isotopic abundances were 8.47 at%, 81.32 at%, and 10.21 at%. respectively, indicating that they contained Fe^2+^ and Fe^3+^. It can be seen that the composite was successfully composed, and the content of Fe_3_O_4_ was much higher than that of MoS_2_.

**Fig. 3:**
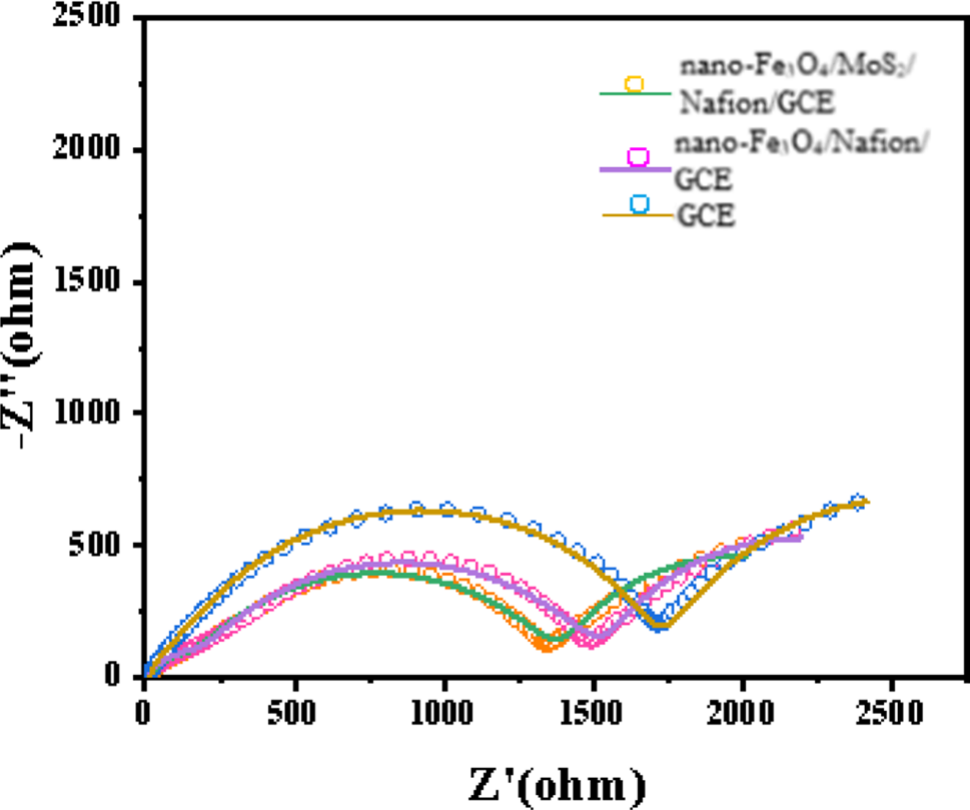
EIS test for nano-Fe_3_O_4_/MoS_2_/Nafion/GCE, nano-Fe_3_O_4_/GCE, and GCE

To further analyze the electrochemical properties of composites, we tested nano-Fe_3_O_4_/MoS_2_ /Nafion/GCE, nano-Fe_3_O_4_/GCE and GCE in 0.1 mol HNO_3_ at EIS response curve as shown in Fig. 3. It can be seen that the arc of nano-Fe_3_O_4_/MoS_2_/Nafion/GCE is the smallest and the arc of GCE is the largest, which means that the modified electrode has stronger electron transfer ability, smaller charge transfer resistance, and the lower the detection limit that can be obtained.

### Electrochemical characterization of Nano-Fe_3_O_4_/MoS_2_/Nafion

**Fig. 4:**
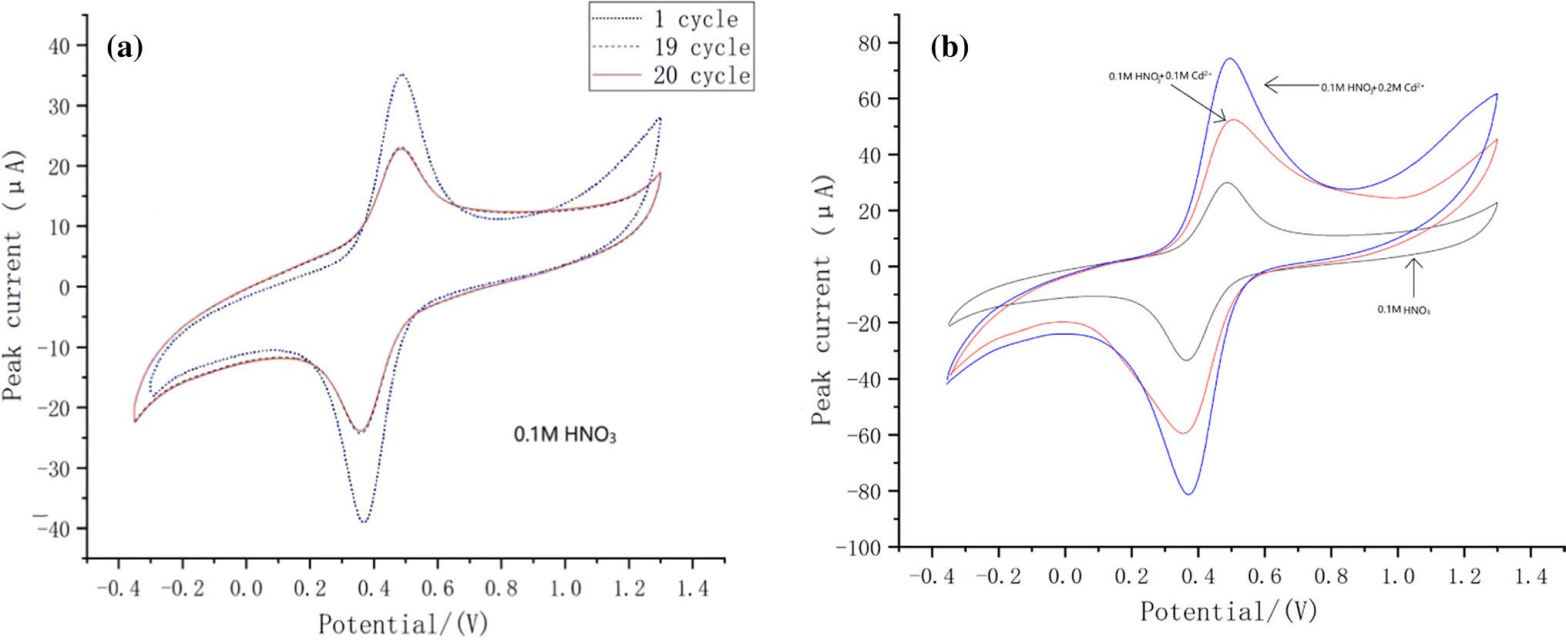
**a** CV curves of Nano-Fe_3_O_4_/MoS_2_/Nafion/GCE for different periods in 0.1 M HNO_3_. **b** CV curves of Nano-Fe_3_O_4_/MoS_2_/Nafion/GCE at different concentrations of Cd^2+^

To detect the electrochemical activity of the modified electrodes, Fig. 4(a) is described separately the 1st, the 19th and the 20th cycles of nano-Fe_3_O_4_/MoS_2_/Nafion modified GCE at 0.1 mol HNO_3_. In the 19th and 20th cycles, it is clear from the figure that the electrochemical activity of nano-Fe_3_O_4_/MoS_2_/Nafion/GCE decreased significantly at first, but it reached a stable state at 19–20 cycles. Figure 4(b) depicts the CV curves of a 0.1 mol/L HNO_3_ solution, compared without adding Cd^2+^ with adding 0.1 mol Cd^2+^ and 0.2 mol Cd^2+^, the redox peak of Cd^2+^ increased with the increase of the concentration of Cd^2+^. It is reasonable to think that this is caused by the increase of Cd^2+^, because the pH of the three solutions is equal, which shows that nano-Fe_3_O_4_/MoS_2_/Nafion/GCE can redox Cd^2+^.

**Fig. 5:**
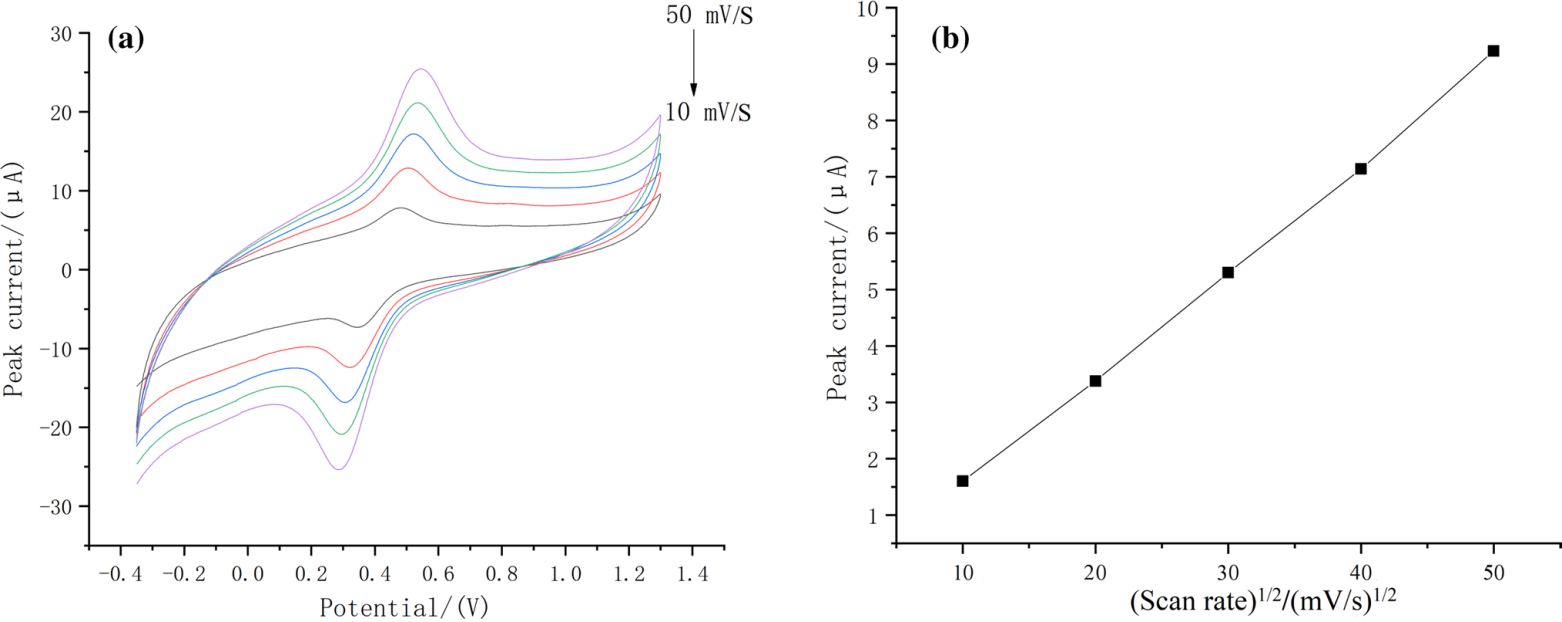
**a** CV curves of Nano-Fe_3_O_4_/MoS_2_/Nafion/GCE at different sweep speeds in 0.1 mol L^−1^ HNO_3_ solution. **b** Standard curve at different sweep speeds

When electrodes are electrochemically reacted, they can be divided into surface process control and diffusion process control, to verify the electrode surface characteristics of Nano-Fe_3_O_4_/MoS_2_/Nafion, Nano-Fe_3_O_4_/MoS_2_/Nafion was placed in a 0.1 mol L^−1^ HNO_3_ solution, and CV tests with different sweep speeds were performed, and the CV diagram shown in Fig. 5(a) was obtained. As the sweep speed increases, the peak current increases, and the peak current Ip has a certain linear relationship with the sweep speed V^1/2^, as shown in Fig. 5(b), indicating that Nano-Fe_3_O_4_/MoS_2_/Nafion is modified. The electrode reaction of the Cd^2+^ sensor is controlled by a diffusion process.

**Fig. 6:**
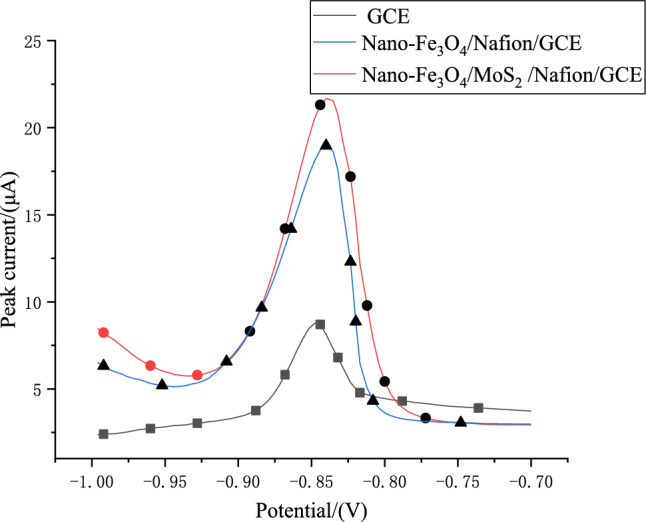
Stripping voltammetric responses of different electrodes in the sodium acetate solution with 250 μg/L Cd^2+^

To compare the effect of different materials on the Cd^2+^ sensor. Figure 6 depicts the DPV detection results of the bare electrode, MoS2/Nafion modified electrode and Nano-Fe_3_O_4_/MoS_2_/Nafion modified electrode in 0.1 mol HAc-NaAc solution under the same conditions. It can be seen from the figure that the response signal of Cd^2+^ on Nano-Fe_3_O_4_/MoS_2_/Nafion/GCE is the strongest. The reason is that because Fe_3_O_4_ has a strong adsorption capacity, it can adsorb Cd^2+^ on the surface of the electrode, and because of the stability of MoS_2_, it can act as a supporting matrix on Fe_3_O_4_ to prevent the phenomenon of agglomeration affecting the detection results. Based on the composite effect of Nano-Fe_3_O_4_/MoS_2_/Nafion, compared with a bare electrode and MoS_2_/Nafion modified electrode, the amount of Cd^2+^ deposited in the modified electrode was greatly increased, and the stripping peak was significantly increased.

### Optimization of electrochemical detection conditions

**Fig. 7:**
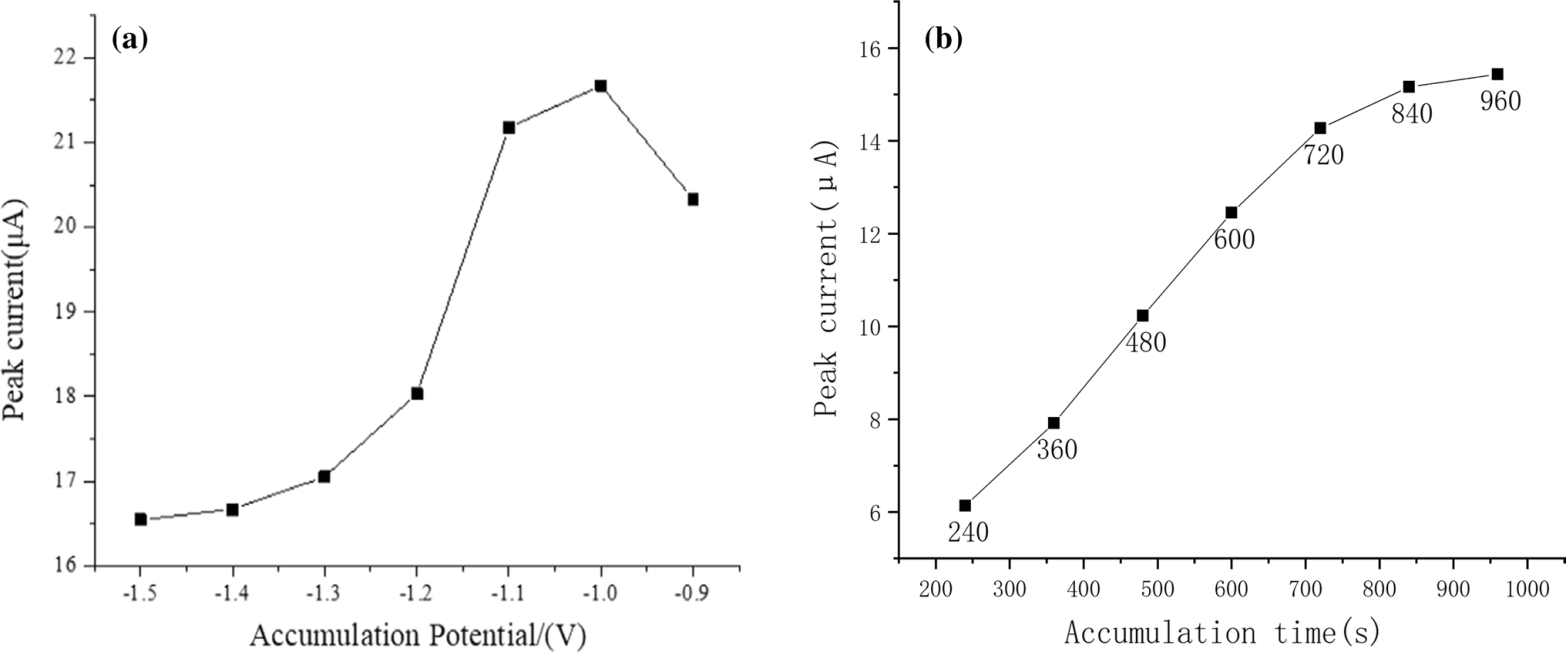
**a** Effect of deposition potential. **b** Effect of deposition time

Considering the effect of deposition potential on Cd^2+^ sensor. It can be seen from Fig. 7(a) that when the potential is − 0.9 V to − 1.0 V, the stripping peak current increases continuously, and when the potential is lower than − 1.0 V, the stripping peak current begins to decrease. The reason is that when the deposition potential is lower than − 1.0 V, a large number of bubbles will appear on the surface of GCE, and these bubbles will not only affect the deposition of Cd^2+^ but also make the deposited Cd^2+^ falls off. When the deposition potential is in the range of − 0.9 V to − 1.0 V, the electrodeposition reaction cannot provide enough electrochemistry to reduce Cd^2+^. Therefore, the optimal deposition potential is -1.0 V.

The effect of different sedimentation times on the dissolution peak needs to be considered afterwards. It can be seen from Fig. 7(b) that when the deposition time is 240–960 s, the peak current increases rapidly with the increase of deposition time, and when it is greater than 720 s, the peak current begins to become stable. The reason is due to limited sites on the electrode surface. Based on the experimental conditions and environmental factors in this experiment, the optimal deposition time is 720 s.

**Fig. 8:**
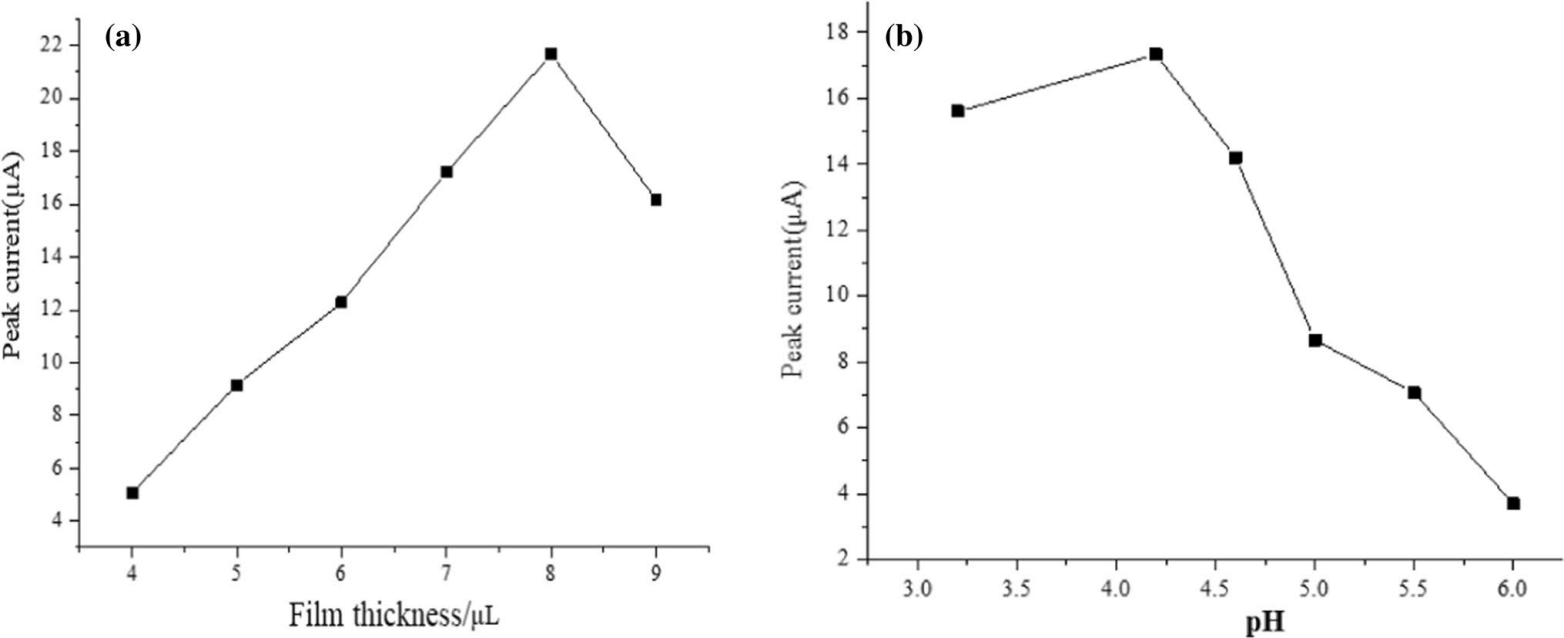
**a** Effect of drop-coating film thickness. **b** Effect of different pH values

Different pH values and different droplet coating amounts can also have significant effects on the Cd^2+^ sensor. It can be seen from Fig. 8(a) that when the drop-coating volume is 5–8 μL, the peak current increases with the increase of deposition time, and when the drop-coating volume exceeds 8 μL, the peak current begins to remain stable. The reason for this phenomenon is as follows: Since the surface area of the electrode is limited when the drop-coating volume is more than 8 μL, the electrode does not have enough surface area to contact with the modified material to deposit Cd^2+^, and the redox reaction of Cd^2+^ will be affected due to too much-modified material. It can be seen that the optimal Drip coating amount is 8 μL. Figure 8(b) compares the effect of different pH values on the stripping peak current. When the pH value is 3.5–4.2, the current increases continuously, but when the pH value exceeds 4.2, the response current begins to decrease. The reasons are as follows: When the pH value is too low, the H^+^ ions in the buffer become more and form a competitive relationship with the Cd^2+^ ions, resulting in hydrogen evolution, which reduces the stripping peak current; When the pH value is too high, the OH^−^ contained in the solution forms a complex reaction with Cd^2+^, which affects the detection of Cd^2+^ sensor. It can be seen that the optimal pH environment condition is 4.2.

**Fig. 9:**
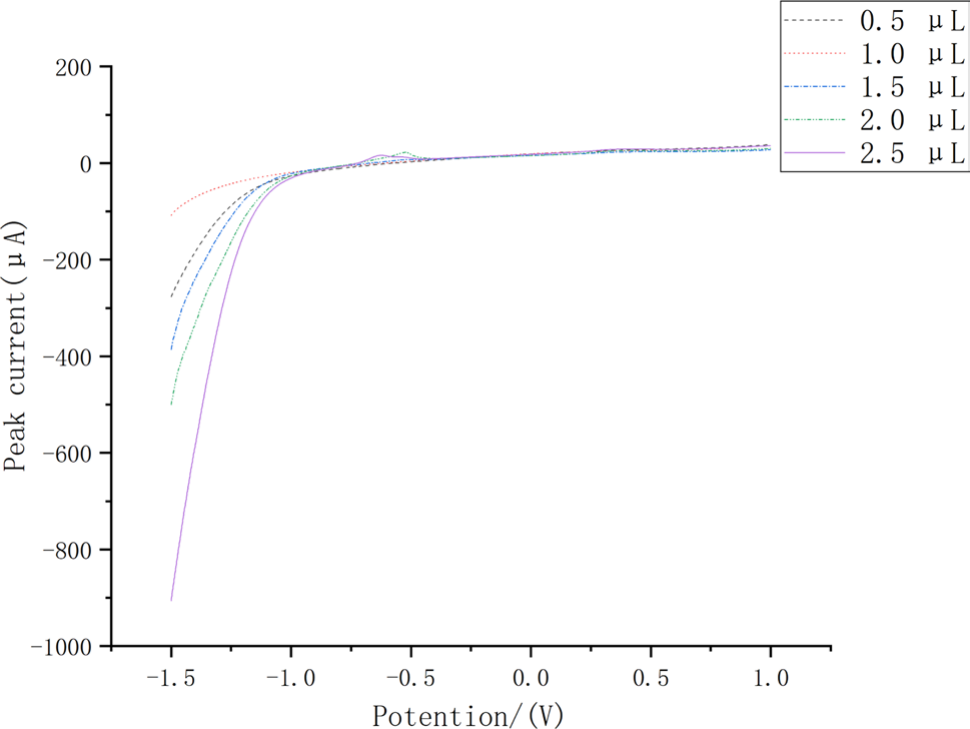
LSV curves with different Nafion content

To detect the effect of different Nafion levels on the sensor, we conducted LSV experiments. Experiments were performed in HAc-NaAc solution at pH = 4.2. Figure 9 shows the LSV curves of Nafion with different contents with an experimental potential of − 0.9 V in HAc-NaAc solution at pH 4.2. As shown in the figure, when the Nafion content increases, the current gradually increases, this shows that when the content of Nafion increases, nafion will produce better electrochemical activity of Nano-Fe_3_O_4_/MoS_2_/Nafion, the optimal content of Nafion in this experiment is determined to be 2.5 μL.

**Fig. 10:**
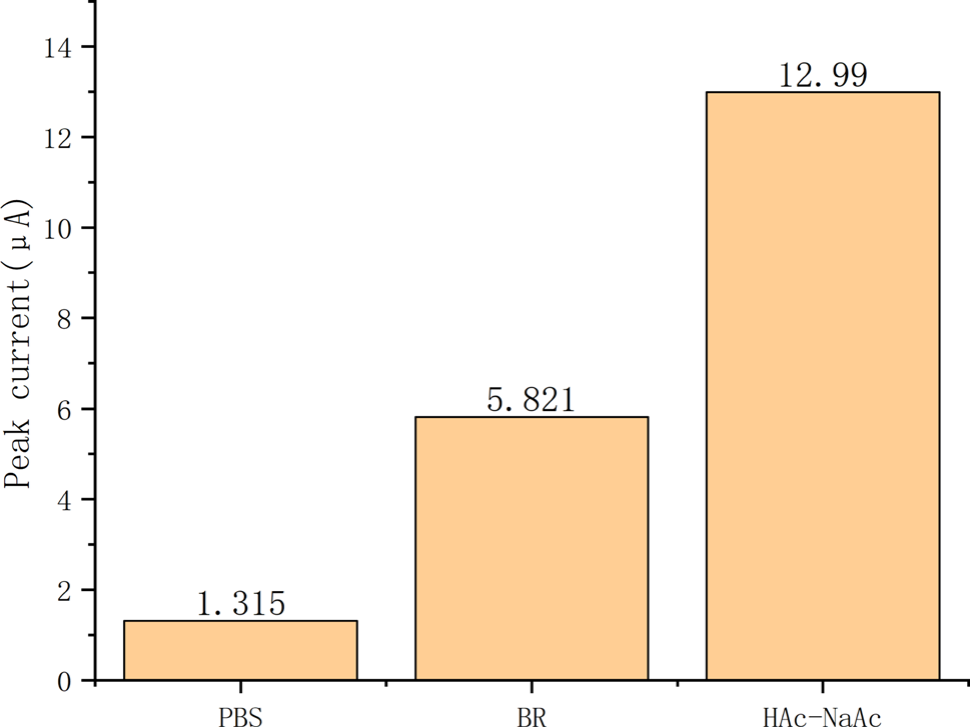
DPV values for different buffers

To investigate the effect of different buffers on the detection of Cd^2+^ sensor, Fig. 10 depicts the DPV values of the modified modified electrodes in the case of different buffers.It can be seen from the figure that the DPV test was performed on Nano-Fe_3_O_4_/MoS_2_ /Nafion/GCE in three different support electrolytes containing 150 μg/L Cd^2+^, and HAc-NaAc was determined as the supporting electrolyte of Cd^2+^ sensor by comparing the values of the histogram.

### Linear standard curve

**Fig. 11:**
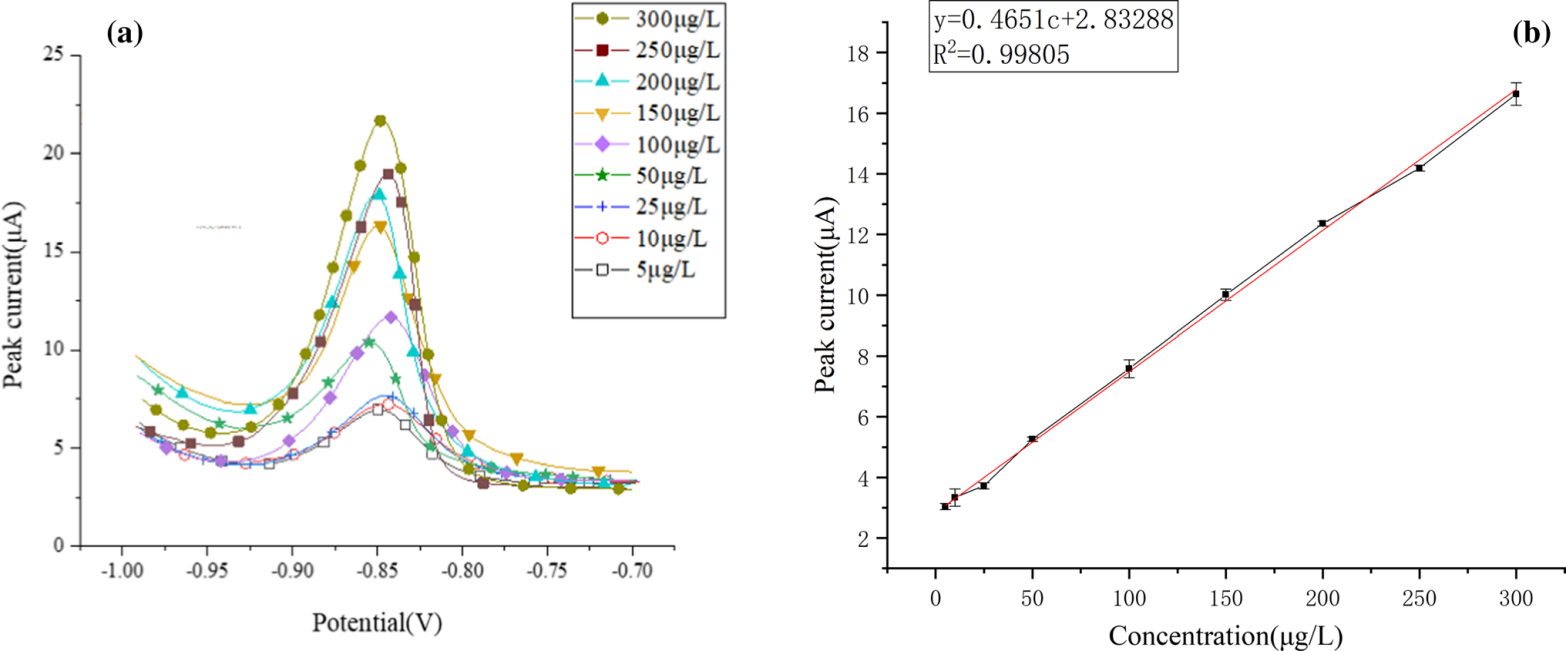
**a** Differential pulsed voltammogram of Cd^2+^ detection on Fe_3_O_4_/MoS_2_/Nafion/GCE. **b** Corresponding linear regression curve

**Fig. 12:**
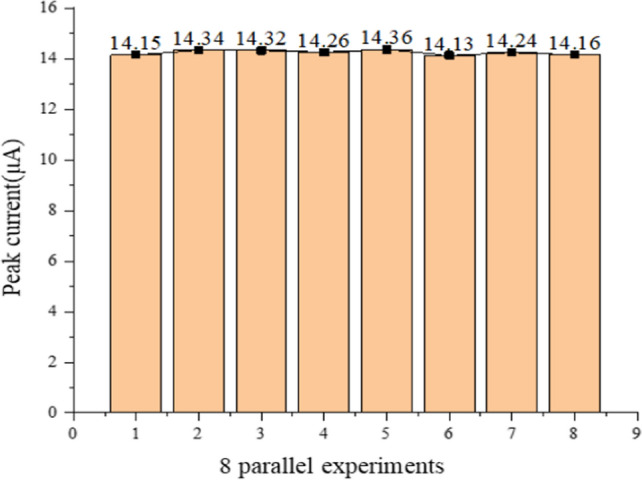
Stability changes of the same modified electrode measured 8 times in 150 μg/L Cd^2+^

According to the above experiments, the optimal deposition potential, the optimal deposition time and other experimental parameters were determined. To obtain the detection of different concentrations of Cd^2+^ by Cd^2+^ sensor, it is necessary to use DPV detection. Figure 11(a) shows the DPV curve for detecting a concentration range of 5–300 μg/L. Each curve is clearly displayed. The Fig. 11(b) is the linear regression curve, In the range of 5–300 μg/L, In the range of 5–300 μg/L, the resulting slope K is 0.4651, by detecting the limit equation:

3 $$ LOD = 3RSD/K $$

4 $$ {\text{RSD}} = \sqrt {\frac{1}{n - 1}\sum\limits_{i = 1}^{n} {n_{i} - \overline{n}^{2} } } $$

where the RSD is the relative standard deviation, K is the slope of the linear equation, and n is the number of detected concentrations. The value of RSD calculated experimentally is 0.82%, The experimental data are shown in Fig. 12. The limit of detection calculated by the experiment is 0.053 μg/L, which is lower than the World Health Organization and the Chinese limit of 5 of Cd^2+^ concentration in drinking water. The experimental results show that Nano-Fe_3_O_4_/MoS_2_ /Nafion/GCE shows good electrochemical performance in Cd^2+^ detection, high sensitivity and low detection limit.

### Stability analysis of modified electrode

In actual use, stability is a very important indicator of the practicality of the sensor. To evaluate the stability of Cd^2+^ sensor in multiple experiments,8 consecutive DPV experiments were carried out in 0.1 mol/L Hac-NaAc buffer with a Cd^2+^ concentration of 250 μg/L. The experimental results are shown in Fig. 12. The RSD of the stripping peak current of 8 DPV experiments was 0.82%, indicating that the Cd^2+^ sensor had good stability.

### Actual sample detection

The water source of the Maowei Sea in Qinzhou City comes from the Qinjiang River and Maoling River as the main runoff into the bay, and its water quality is complex. The analysis results of the investigation of water quality and sediment environmental elements in the adjacent waters of the mouth of Maowei Sea in Qinzhou are listed in Table 1. In the table, “nd” is not checked out. The units of suspended solids, DO, COD, inorganic nitrogen and phosphate are “mg/L”, and the units of Hg, Cd, Pb, As, Cu and Zn are “μg/L”. The collection, preservation and analysis of samples are carried out in accordance with the corresponding requirements in the Code for Marine Survey (GB/T 12763-2007) and the Code for Marine Monitoring (GB17378-2007).

**Table 1:** Statistical table of analysis results of water quality and environmental elements in sea area

Stations	PH	Suspended solids	DO	COD	Hg	Cd	Pb	As	Cu	Zn	Inorganic nitrogen	Phosphate
Maximum	7.25	56	6.74	3.22	0.017	2.209	0.991	0.84	10.3	8.65	0.786	0.054
Minimum	7.00	22	5.18	1.78	0.009	0.902	0.548	nd	7.14	4.76	0.561	0.035
Average value	7.15	36.5	5.94	2.58	0.012	1.556	0.712	0.34	8.68	6.83	0.630	0.047

The water samples were taken from different areas in the Maowei Sea. After the samples were obtained, the samples were left for 48 h. The supernatant liquid of the water samples was filtered with a 0.45 μm filter membrane, and a 50 mL water sample was taken by graduated cylinder for spike-and-recovery experiment. It can be seen from Table 2 that the Cd^2+^ concentration of the actual water sample is lower than the national limit of 5 μg/L in drinking water, and the recovery rates of the spike-and-recovery experiments are 100.2%, 102.7% and 99.4%, respectively, indicating that the detection accuracy of this modified material is high and the Cd^2+^ sensor can be used for high-sensitivity experiments of real water samples.

**Table 2:** Detection of Cd^2+^ concentration in actual samples

Sample	Measured value (μg/L)	Spike value (μg/L)	Total measured value (μg/L)	Recovery rate (%)
Sample 1	1.226	20	21.2610	100.2
Sample 2	1.348	20	21.9710	102.9
Sample 3	2.321	25	27.129	99.2

To further evaluate the electrochemical performance of The Cd^2+^ sensor, the results were compared with other results based on the detection of cadmium ions in different materials modified GCEs. The results are shown in Table 3. As can be seen from the table, Nano-Fe_3_O_4_/MoS_2_/Nafion/GCE has a lower detection limit and a wider linear range than some other composites that require multi-step preparation. The results show that Nano-Fe_3_O_4_/MoS_2_/Nafion/GCE has high sensitivity, fast response and good stability, and can be applied to the detection of cadmium ion in seawater not lower than ppb level.

**Table 3:** Comparison of Cd^2+^ performance analysis of different electrochemical sensors

Electrode	Sensitivity	Detection limit	References
Nafion/Bi/NMC/GCE	2–100 μg L^−1^	1.5 μg L^−1^	[21]
BiOCl/MWCNTs/GCE	5–50 μg L^−1^	4.0 μg L^−1^	[22]
CNPs/BiNPs/SPCE	1–50 μg L^−1^	1.5 μg L^−1^	[23]
Bismuth/MXene/GCE	9–90 μg L^−1^	1.39 μg L^−1^	[24]
BiNPs@NPCGS/GCE	0.08–0.8 mm	4.1 nm	[25]
Bi/C/GCE	1–100 μg L^−1^	0.81 μg L^−1^	[26]
g-CN/SnONPs	0.05–100 μg L^−1^	0.16 μg L^−1^	[27]
SnO/NPs/LIG	0.1–160 μg L^−1^	0.01 μg L^−1^	[28]
Nano-Fe_3_O_4_/MoS_2_/Na-fion/GCE	5–300 μg L^−1^	0.053 μg L^−1^	This paper

## Conclusion

In this paper, Nano-Fe_3_O_4_/MoS_2_/Nafion/GCE was prepared and a Cd^2+^ sensor was constructed. The optimal conditions were determined: In 0.1 mol/L HAc-NaAc solution, the solution pH was 4.2, deposition potential was − 1.0V, and the deposition time was 720 s. After optimization, it was found that the response peak current of Cd^2+^ showed a good linear relationship with the concentration in the range of 5–300 μg/L and the LOD of 0.053 μg/L. Finally, the coastal seawater was collected for spike-and-recovery experiments. The experimental results show that the recovery rate of Cd^2+^ is 99.4% to 102.7%. Based on the above conclusions, this improved electrode can better detect Cd^2+^ in seawater.

## Data Availability

Data are available on request to the authors.
